# Time-Course Responses of Muscle-Specific MicroRNAs Following Acute Uphill or Downhill Exercise in Sprague-Dawley Rats

**DOI:** 10.3389/fphys.2019.01275

**Published:** 2019-10-02

**Authors:** Xin Yin, Yan Zhao, Yi Li Zheng, Jin Zhi Wang, Wei Li, Qiu Ju Lu, Qiang Nian Huang, Chen Yu Zhang, Xi Chen, Ji Zheng Ma

**Affiliations:** ^1^State Key Laboratory of Pharmaceutical Biotechnology, Collaborative Innovation Center of Chemistry for Life Sciences, Jiangsu Engineering Research Center for MicroRNA Biology and Biotechnology, NJU Advanced Institute for Life Sciences (NAILS), School of Life Sciences, Nanjing University, Nanjing, China; ^2^The Research Center of Military Exercise Science, The Army Engineering University of PLA, Nanjing, China; ^3^Department of Exercise and Heath, Nanjing Sports Institute, Nanjing, China

**Keywords:** downhill exercise, uphill exercise, muscle-specific microRNAs, plasma, exosomes

## Abstract

**Objective:** The physiological characteristics and acute responses underpinning uphill running differ from those of downhill running and remain less understood. This study aimed to evaluate time-course changes of muscle-specific microRNA (miRNA) responses in striated muscle or circulation in response to uphill and downhill running.

**Methods:** Male Sprague-Dawley rats (*n* = 84) were randomly assigned to a sedentary group (*n* = 12) and an exercise group (*n* = 72). The exercise group performed 90 min of uphill or downhill running. The striated muscle (quadriceps, gastrocnemius, soleus, and cardiac muscle) or circulation (plasma, exosome, exosome-free) levels of six muscle-specific miRNAs (miR-1, miR-133a, miR-133b, miR-206, miR-208a, and miR-499) were assessed at rest, immediately following exercise, and during recovery (1 h and 48 h).

**Results:** Our results show that miR-1 and miR-133a levels are both decreased in quadriceps following downhill running (*p* < 0.05) while there is no change after uphill running (*p* > 0.05). In gastrocnemius, both uphill and downhill running decreased miR-1 level immediately after exercise and returned to baseline during recovery (*p* < 0.05): interestingly, only miR-499 significantly increased following uphill running (*p* > 0.05). Of the cell-free miRNAs in circulation, only the miR-133b levels in plasma were not affected following uphill running (*p* > 0.05); the other miRNA levels significantly increased immediately after exercise (*p* < 0.05), decreased at 1 h and significantly increased at 48 h after exercise (*p* < 0.05). All selected miRNA levels in exosomes were not affected following uphill running (*p* > 0.05), while all selected miRNA levels significantly increased during early recovery after downhill running (*p* > 0.05). In addition, only the miR-133a level in the exosome-free condition showed significant changes following uphill running (*p* < 0.05), while miR-1, miR-133a, and miR-499 levels showed significant changes after downhill running (*p* < 0.05).

**Conclusion:** The results indicate that miRNA undergoes dynamic changes in tissue may play an important role in regulating different stress/adaptation following uphill and downhill running. It is likely that changed miRNA levels in plasma may act as a new biomarker for monitoring whole muscular stress during recovery.

## Introduction

Exercise is a form of physiological stress that has a marked effect on the muscle system (Egan and Zierath, 2013). Many daily activities require muscles to perform concentric/eccentric contractions. It is generally known that unfamiliar exercise, especially involving forceful eccentric muscle contractions, can result in temporary, repairable skeletal muscle damage (Ebbeling and Clarkson, 1989), such as increases in creatine kinase and delayed onset of muscle soreness that peaks 36–72 h after the exercise bout (Bird et al., 2014). It should be noted that “pure” uphill or downhill running (muscle shortening or lengthening) has several exercise-related intrinsic features compared with the level running (Giandolini et al., 2016). Thus, uphill or downhill running is often used as a model of concentric/eccentric exercise in rats to examine skeletal muscle function/damage processes following concentric/eccentric-induced mechanical stress (Munehiro et al., 2012). It is suggested that several grade-specific differences exist between level, uphill, and downhill running regarding biomechanics, neuromuscular adaptations and physiological responses (Vernillo et al., 2017). Because eccentric exercise offers a promising training modality to enhance performance and to prevent injuries in clinical settings, it is important to precisely determine skeletal muscle adaptation processes underpinning these different training stimuli (Assumpcao Cde et al., 2013).

Recently, microRNAs (miRNAs), or small non-coding RNA molecules, which are involved in a variety of basic biological processes that negatively modulate gene expression, have been recognized as important exercise-induced regulatory molecules (Franchi et al., 2017). The miRNAs that have been implicated in exercise-induced skeletal muscle remodeling (McCarthy and Esser, 2007; Kirby and McCarthy, 2013; Masi et al., 2016) include eight muscle-specific or enriched miRNAs (myomiRs): miR-1, miR-133a, miR-133b, miR-206, miR-208a, miR-208b, miR-486, and miR-499 (Horak et al., 2016). In addition, given that skeletal muscle (approximately 40% of body weight) undergoes contraction and causes major disruption to metabolic homeostasis, it is not surprising that the majority of exercise-induced secreted molecules originate from skeletal muscle (Egan and Zierath, 2013). More importantly, the discovery of cell-free miRNAs in serum, plasma, and other body fluids has yielded an invaluable potential source of non-invasive biomarkers for exercise (Mooren et al., 2014; Sapp et al., 2017). Experimental evidence indicates that four potential forms of miRNAs are released from cells (Ortiz-Quintero, 2016): miRNAs encapsulated within exosomes (Valadi et al., 2007), complexed with Argonaute2 protein (Ago2) (Arroyo et al., 2011), bound to high-density lipoprotein (HDL) (Vickers et al., 2011), or bound to the RNA-binding protein, nucleophosmin (NPM1) (Wang et al., 2010). Recently, evidence has suggested that during exercise, exosomes can be released into circulation, which may mediate the beneficial effects of exercise (Fruhbeis et al., 2015; Whitham et al., 2018).

At present, muscle-specific microRNA responses to uphill and downhill running remain unknown; thus, we aimed to verify whether these miRNAs differences exist in response to concentric versus eccentric actions. Based on the distinct characteristics of eccentric muscle actions, we hypothesize that during downhill (high eccentric component) and uphill (high concentric component) running, the time-course changes of muscle-enriched miRNAs in striated muscle or plasma may induce specific physiological responses, which may help to better understand the two distinct loading stimuli.

## Materials and Methods

### Animals

Male Sprague-Dawley rats (*n* = 84), 8 weeks old, were purchased from Sino-British SIPPR/BK Lab Animal Ltd. Rats were housed in cages in a temperature- and humidity-controlled room and maintained on a 12-h light–dark cycle with food and water *ad libitum*. All rats were habituated to our treadmill (speed = 5 m/min; time = 5 min; grade = 0°) for a period of 5 days and kept as sedentary for another 2 days prior to formal experimental intervention. All experiments were performed between 1 and 6 PM. The procedures for care and use of animals strictly followed the *Guide for the Care and Use of Laboratory Animals* published by the US National Institutes of Health (NIH Publication N. 85–23, revised 1996). The protocol was approved by the Ethics Committee of Nanjing University.

### Experimental Design

**Figure 1:**
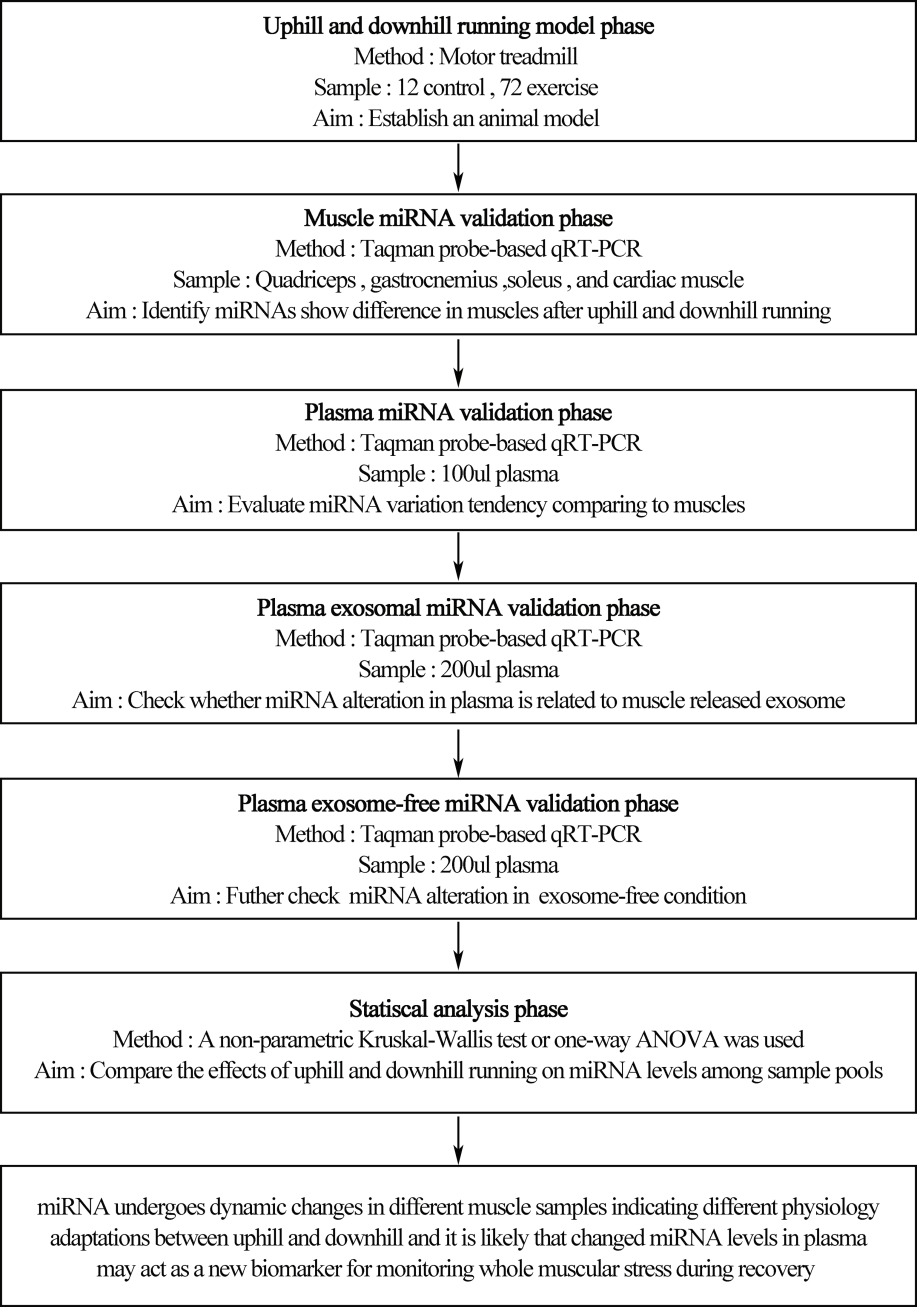
Treadmill running program. Rats underwent an acute bout of uphill and downhill running for 90 min that consisted of a speed of 20 m/min and a grade of either −15 or 15%. Blood samples, quadriceps, gastrocnemius, soleus, and cardiac muscles were collected before exercise (rest), immediately after exercise (0 h) and during recovery (1 and 48 h) of uphill and downhill running. Both groups included four time points, and each time point consisted of 12 rats.

**Figure 2:**
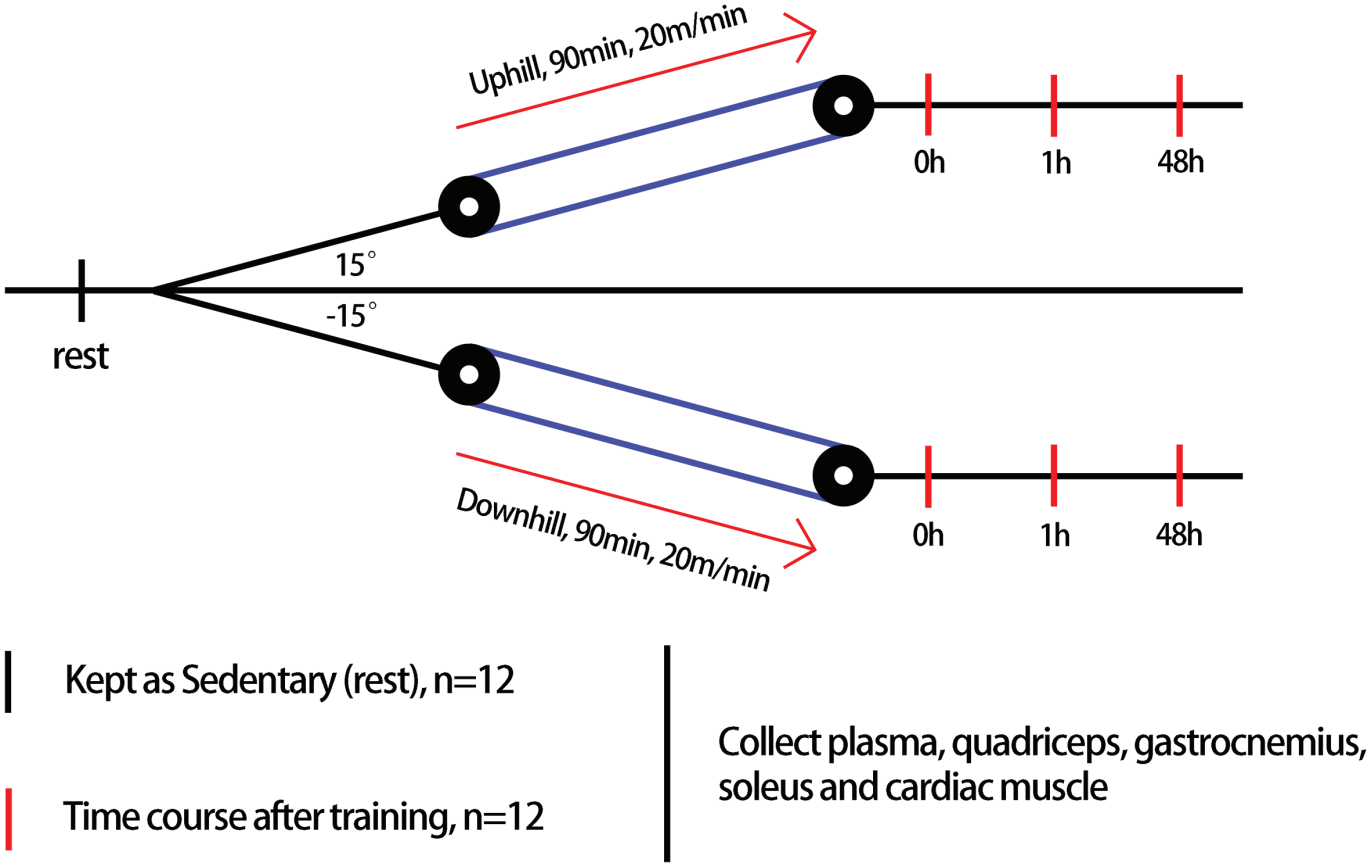
An overview of the experimental design.

Rats were randomly assigned to the sedentary group (rest, *n* = 12) or exercise group (*n* = 72) (Figure 1). The exercise group underwent treadmill (ANHUI ZHENGHUA BIOLOGIC APPARATUS FACILITIES CO., LTD.) running on different modalities of uphill running (mainly concentric factor, *n* = 36) and downhill running (mainly eccentric factor, *n* = 36). The downhill run consisted of running at 20 m/min at a grade of 15% for 90 min based on a model found to induce significant injury to skeletal muscle with some modification (Chavanelle et al., 2014). The uphill run was performed with the same conditions as the downhill run, except the grade was altered to ensure a matched running distance (i.e., 90 min, 20 m/min, grade of −15%). For each experimental session, we randomly choose four rats from different cages to perform the experiment at one time to avoid differences between batches. Three rats underwent a bout of treadmill exercise, and the last one was treated with rest without a running exercise. A 5-min warm-up and cool-down at 5 m/min were also included. Blood samples, quadriceps, gastrocnemius, soleus, and cardiac muscle were collected before exercise (rest), immediately after exercise (0 h), and during recovery (1 and 48 h) from uphill and downhill running. Both the uphill and downhill run included four time points, and each time point consisted of 12 rats (Figure 2).

### Blood Samples

During each exercise experiment, 4 ml of blood was drawn from the abdominal venous at different time points after exercise in standard anticoagulant (EDTAK2)-treated vacutainer tubes. Blood samples were centrifuged at 1,500× g for 10 min immediately after each blood draw to pellet cellular elements first and then centrifuged at 10,000 × *g* for 5 min at 4°C to remove cellular debris entirely. The supernatant plasma was used for plasma, exosomal, and exosome-free miRNA analyses. Exosomes were isolated from 200 μl of plasma using ExoQuick™ Exosome Precipitation Solution (SBI) according to the manufacturer’s instructions. In brief, 200 μl of plasma was diluted with 50 μl of SBI solution and mixed well by inverting the tube softly. The tube was refrigerated for 30 min without rotating during the incubation period. The mixture was centrifuged at 1,500 × *g* at 4°C for 30 min. The exosomes appeared as a white pellet at the bottom of tube, and the supernatant was transferred into another tube and retained for the analysis of the exosome-free miRNA analysis. Then, the tube containing the exosomes was centrifuged at 1,500 × *g* for 5 min to remove the supernatant entirely. All samples were immediately frozen at −80°C.

### Muscle Samples

All rats were euthanized by cervical dislocation after blood was drawn, and the quadriceps, gastrocnemius, soleus, and cardiac muscles (apex of heart) were harvested into liquid nitrogen, snap frozen, and stored at −80°C for subsequent analyses.

### Selection of Candidate miRNAs

Six muscle-specific miRNAs (miR-1, miR-133a, miR-133b, miR-206, miR-208a, and miR-499) were selected for this study, which are abundant in cardiac or skeletal muscles (Horak et al., 2016).

### Total RNA Isolation and Quantification of miRNAs

RNA was isolated from 100 μl of plasma using a one-step phenol/chloroform (Thermo fisher scientific) purification protocol, as reported previously (Liu et al., 2011). For the muscle and exosomal RNA extraction, TRIzol regent (Takara) was used to extract RNA from 100-mg muscle sample, the pellet of exosomes from 200 μl of plasma, and the exosome-free supernatant. Samples from four time points were processed in the same batch in order to avoid differences caused by operation in the extraction process.

Hydrolysis probe-based RT-qPCR was carried out using a TaqMan PCR kit and a Roche LightCycler 480 II. The cycle threshold (Ct) data were used to show the results and were calculated from triplicate PCRs. The relative expression of miRNAs in muscles was normalized to Rnu6, and Ct values were calculated using the 2^−ΔΔCt^ method. ΔCt was calculated by subtracting the Ct values of Rnu6 from the average Ct values of the target miRNAs. ΔCt values were then compared (ΔΔCt) with the baseline at the rest time point (normalized to a fold-change of 1). The absolute concentration of miRNAs in the plasma, exosome, and exosome-free supernatant was calculated by referring to calibration curves developed with the corresponding synthetic miRNA oligonucleotides. Both the uphill and downhill groups shared the same rest group, and data are expressed as fold-difference compared to the rest group.

### Statistical Analysis

GraphPad Prism 5 was used to analyze the data. Data are presented as the means ± standard errors of the mean (SEM). All miRNA levels at baseline (rest) were assigned a fold-change of 1. The normality of the data distribution was tested using the Kolmogorov–Smirnov test. In addition, a non-parametric Kruskal-Wallis test or one-way ANOVA for unpaired values was used for the plasma, exosome, exosome-free supernatant and muscles. When appropriate, a Bonferroni multiple comparison or a Dunn multiple comparison *post hoc* test was applied to compare groups at different time points. Values of *p* < 0.05 were considered significant.

## Results

### Time-Course Changes of Six Muscle-Specific miRNA Levels in Quadriceps, Gastrocnemius, Soleus, and Cardiac Muscle in Response to Uphill and Downhill Running

**Figure 3:**
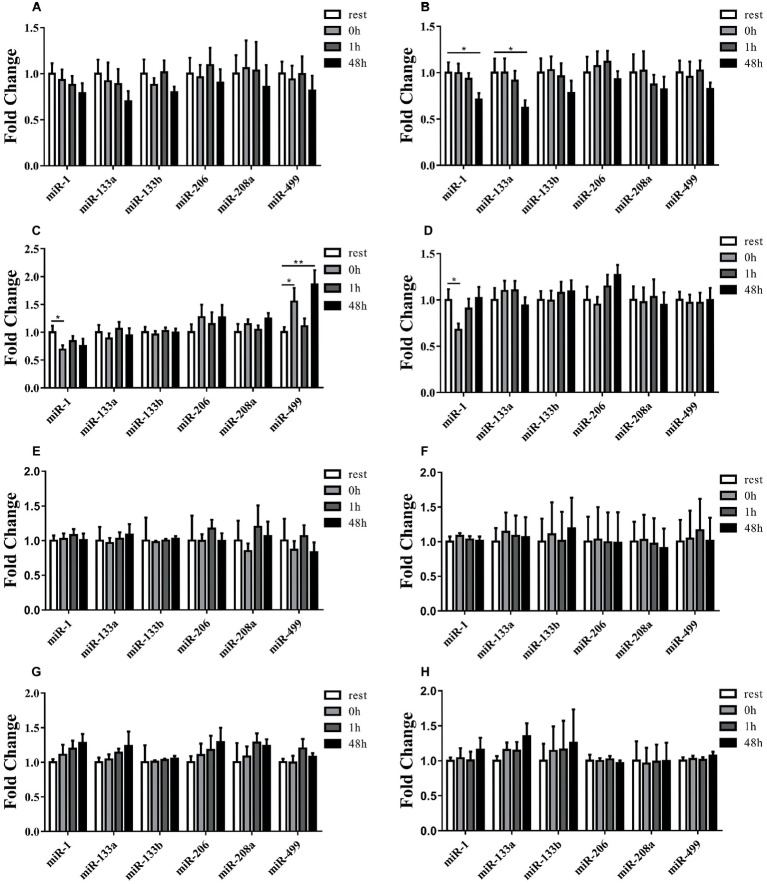
Time-course changes of miR-1, miR-133a, miR-133b, miR-206, miR-208a, and miR-499 levels in quadriceps, gastrocnemius, soleus, and cardiac muscle in response to uphill **(A,C,E,G)** and downhill running **(B,D,F,H)** (*n* = 12 per time point). Both uphill and downhill running shared the same rest group, and data are expressed as the fold-difference compared to the rest group. miRNA expression in muscles is normalized to Rnu6. *denotes *p* < 0.05; **denotes *p* < 0.01.

A schematic scheme was conducted to summarize the whole ideal of the experiment (Figure 1). In the initial stage, we measured miRNA levels in striated muscle in order to identify whether uphill and downhill running affect miRNA levels in muscle differently. Following the uphill run, only the miR-1 levels in the gastrocnemius decreased immediately following exercise, and the miR-499 levels significantly increased immediately following exercise or 48 h following exercise compared to those of the rest condition (*p* < 0.05) (Figures 3A,C,E,G). Following the downhill run, only the miR-1 and miR-133a levels in the quadriceps significantly decreased at 48 h compared to those of the rest condition (*p* < 0.05). The miR-1 level in the gastrocnemius decreased immediately following exercise and returned to the baseline level at 1 h, while the other muscle-enriched miRNA levels did not show significant changes at different time points (*p* > 0.05). In addition, all selected specific miRNA levels in the soleus and cardiac muscles did not show significant changes at different time points (*p* > 0.05) (Figures 3B,D,F,H).

### Time-Course Changes of Six Muscle-Specific miRNA Levels in Plasma in Response to Uphill and Downhill Running

**Figure 4:**
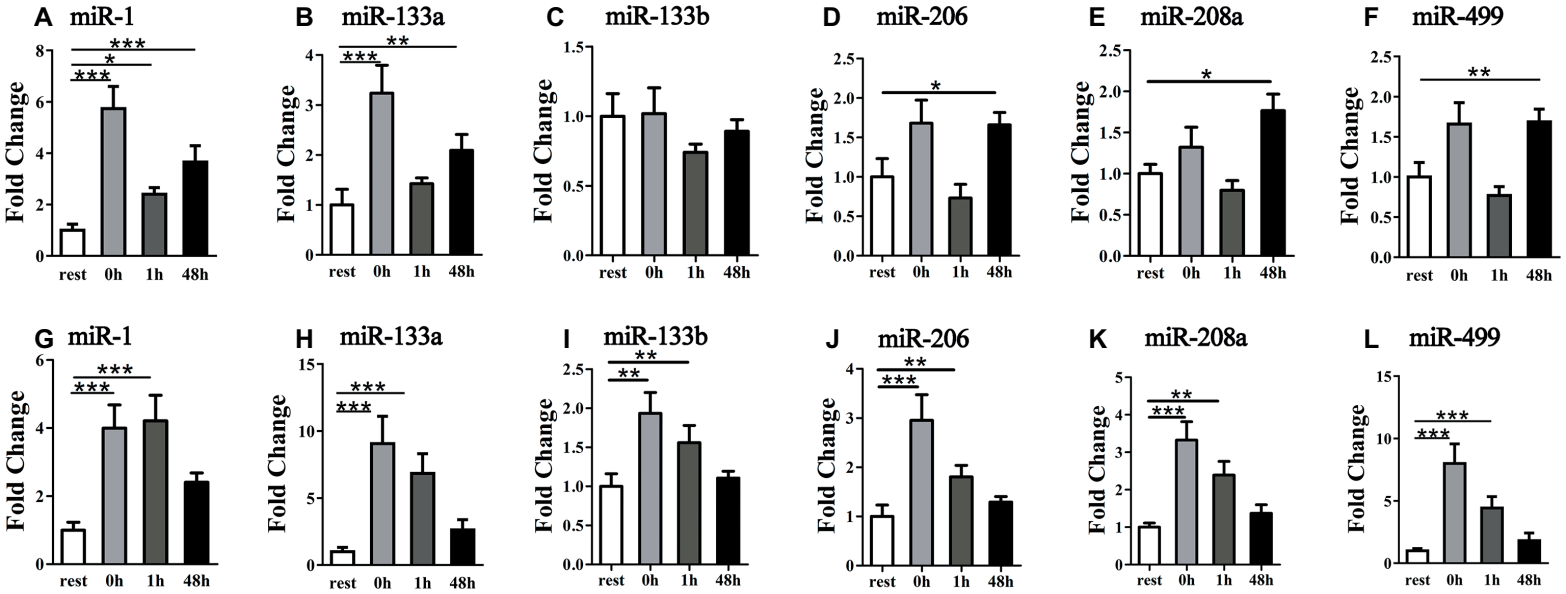
Time-course changes of miR-1, miR-133a, miR-133b, miR-206, miR-208a, and miR-499 levels in plasma in response to uphill **(A–F)** and downhill **(G–L)** running (*n* = 12 per time point). Both uphill and downhill running shared the same rest group, and data are expressed as the fold-difference compared to the rest group. The absolute concentration of miRNAs was calculated by referring to calibration curves developed with the corresponding synthetic miRNA oligonucleotides. *denotes *p* < 0.05; **denotes *p* < 0.01; ***denotes *p* < 0.001.

To examine the relationship of miRNA alteration between muscle and plasma, we measured miRNA levels in plasma. Following the uphill run, the miR-133b levels in the plasma did not show significant changes at different time points (*p* > 0.05), but the other muscle-enriched miRNA levels at 0 h significantly increased compared to those of the rest condition (*p* < 0.05), decreased at 1 h and significantly increased 48 h after exercise (*p* < 0.05) (Figures 4A–F). Following the downhill run, all selected miRNA levels in the plasma at 0 and 1 h significantly increased (*p* < 0.05) compared to those of the rest condition and returned to the baseline level 48 h after exercise (*p* > 0.05) (Figures 4G–L).

### Time-Course Changes of Six Muscle-Specific miRNA Levels in Exosomes in Response to Uphill and Downhill Running

**Figure 5:**
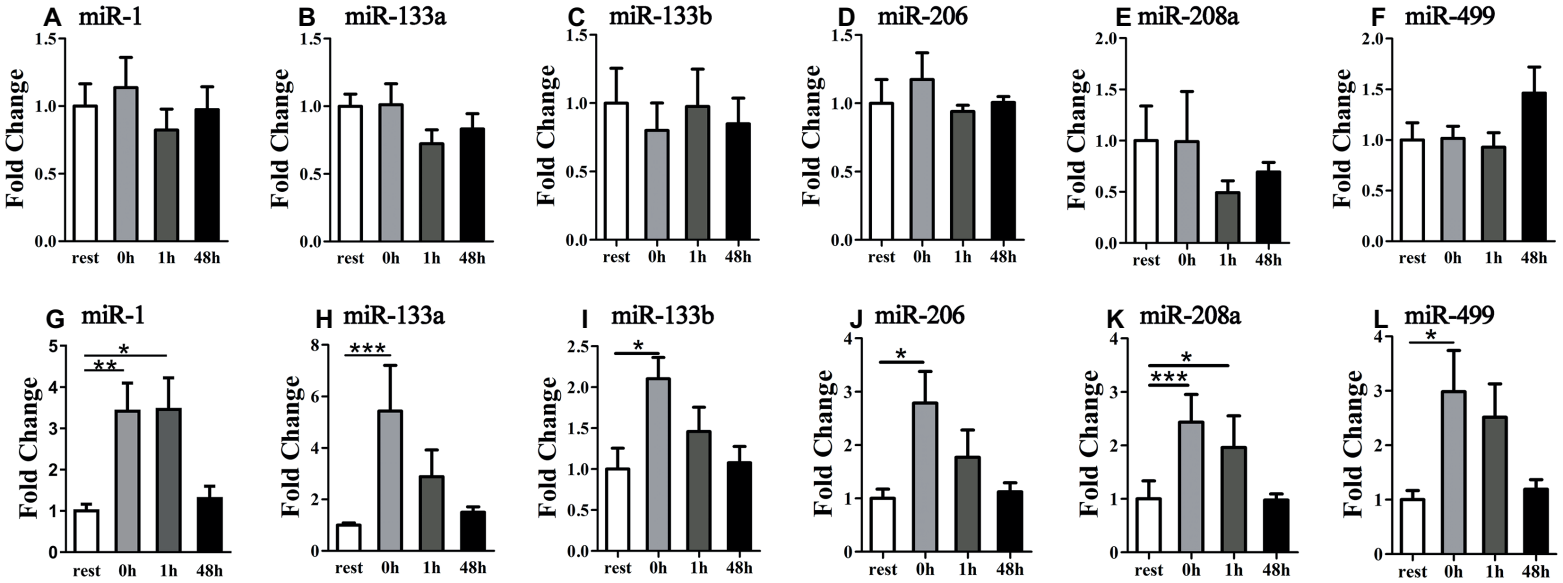
Time-course changes of exosomal miR-1, miR-133a, miR-133b, miR-206, miR-208a, and miR-499 levels in plasma in response to uphill **(A–F)** and downhill **(G–L)** running (*n* = 12 per time point). Both uphill and downhill running shared the same rest group, and data are expressed as the fold-difference compared to the rest group. The absolute concentration of miRNAs was calculated by referring to calibration curves developed with the corresponding synthetic miRNA oligonucleotides. *denotes *p* < 0.05; **denotes *p* < 0.01; ***denotes *p* < 0.001.

To check whether miRNA alteration in plasma is related to muscle-released exosome, we measured miRNA levels in exosome extracted from plasma. Following the uphill run, all selected miRNA levels did not show significant changes at different time points (*p* > 0.05) (Figures 5A–F); following the downhill run, all selected miRNA levels in the exosomes at 0 or 1 h significantly increased (*p* < 0.05) compared to those of the rest condition and returned to the baseline level 48 h after exercise (*p* > 0.05) (Figures 5G–L).

### Time-Course Changes of Six Muscle-Specific miRNA Levels in Exosome-Free Supernatant in Response to Uphill and Downhill Running

**Figure 6:**
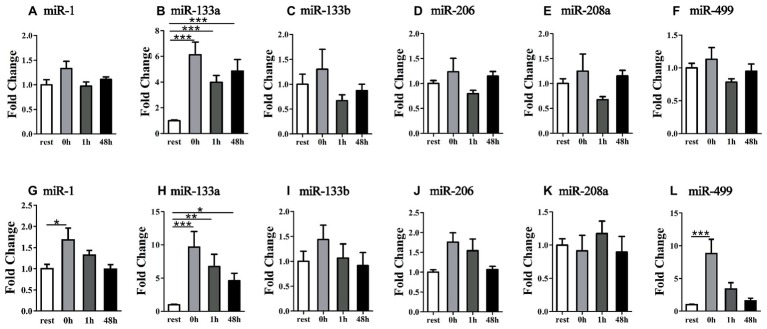
Time-course changes of exosome-free miR-1, miR-133a, miR-133b, miR-206, miR-208a, and miR-499 levels in plasma in response to uphill **(A–F)** and downhill **(G–L)** running (*n* = 12 per time point). Both uphill and downhill running shared the same rest group and data are expressed as the fold-difference compared to rest. The absolute concentration of miRNAs was calculated by referring to calibration curves developed with the corresponding synthetic miRNA oligonucleotides. *denotes *p* < 0.05; **denotes *p* < 0.01; ***denotes *p* < 0.001.

To further explore the existence form of selected muscle-specific miRNA, we measured miRNA levels in exosome-free condition. Following the uphill run, only the miR-133a showed significant changes at different time points (*p* < 0.05) (Figures 6A–F). Following the downhill run, the exosome-free miR-1 and miR-499 levels at 0 and 1 h significantly increased (*p* < 0.05) compared to those of the rest condition, and returned to the baseline level at 48 h after exercise (*p* > 0.05), and the miR-133a levels were significantly increased at different time points (*p* < 0.05). In addition, the other selected miRNA levels did not show significant changes at different time points (*p* > 0.05) (Figures 6G–L).

## Discussion

The metabolic overload and mechanical strain have been regarded as the main factors generating muscle damage after uphill or downhill exercise (Assumpcao Cde et al., 2013). Overall, the results showed that the time-course changes in the muscle-specific miRNA profile are associated with several acute differences in physiological responses to eccentrically emphasized exercise in comparison to concentric exercise, mainly including various parts of muscle tissue, different time-course of changes in plasma, and exosome or exosome-free conditions.

It is well known that unaccustomed uphill or downhill exercise has been shown to produce muscle damage during the post-exercise period (Bird et al., 2014). In the context of same running speed, the eccentric exercise has a lower oxygen uptake than concentric exercise (Minetti et al., 2002). Interestingly, eccentric exercise increases the energy consumption in a resting state 72 h after exercise (Hackney et al., 2008). In addition, the activation of muscle satellite cells after exercise is closely related to muscle repair and reconstruction during recovery (Fry et al., 2017). Also, the degree of activation of satellite cells is related to the mode of exercise. Hyldahl et al. found that eccentric exercise can activate satellite cells to a greater extent, resulting in greater exercise adaptation (Hyldahl et al., 2014). The time course of the events involves disturbance in metabolic function/damage in components of excitation-contraction system and sarcomeres and degeneration and regeneration of muscle fibers (Qaisar et al., 2016).

At present, the precise mechanisms underpinning the unique physiological characteristics during uphill or downhill exercise are not well understood (Isner-Horobeti et al., 2013; Vogt and Hoppeler, 2014; Douglas et al., 2017; Franchi et al., 2017; Vernillo et al., 2017).

Exercise induces epigenetic changes through several mechanisms including chromatin structure changes (methylation or histone acetylation), DNA methylation, and miR expression. These mechanisms modulate, positively or negatively, expressions of the genes related to different exercise-induced stress/adaptive processes (Masi et al., 2016). MyomiRs represent an integral part of skeletal muscle development with a significant influence on muscle metabolism during quiescence, proliferation, differentiation, and regeneration (Horak et al., 2016). Previous studies have suggested that miRNAs also play an important role in mediating the response and adaptation of skeletal muscle to various modes of exercise (Kirby and McCarthy, 2013; Russell et al., 2013). For example, the four myomiRs (miR-1, miR-133a, miR-133b, and miR-206) are negatively modulated in the human vastus lateralis muscle after 12 weeks of endurance exercise (McCarthy and Esser, 2007). In the present study, uphill running down-regulated miR-1 level and up-regulated miR-499 level over the time course in gastrocnemius; while eccentric exercise down-regulated miR-1 and miR-133a in quadriceps or gastrocnemius over the time course, but none of the miRNA levels changed in soleus muscle. Pervious study has shown that gastrocnemius and soleus are more recruited during uphill running while quadriceps are more often used during downhill running (Isner-Horobeti et al., 2014). Interestingly, our results show that only miR-1 and miR-499 levels changed in gastrocnemius following uphill running but not in quadriceps, which indicates that in addition to exercise modality, different exercise load of different muscle parts may also affect the levels of miRNA. Moreover, the four miRNAs down-regulation was associated with improved endurance capacity, VO_2max_, and insulin sensitivity. Studies with predicted target genes appointed out Cdc-42 and Erk-1/2, two proteins involved in the activation of MAPK signaling pathway that controls different processes, including muscle growth, repair, and remodeling (McCarthy and Esser, 2007). Quadriceps and gastrocnemius are mainly consisted of fast muscle fiber and endurance can convert fast muscle to slow muscle and soleus is mainly consisted of slow muscle. Accordingly, this may provide evidence that why there is no miRNA levels changed after both exercise modality in soleus and miR-1/133a level variation in quadriceps or gastrocnemius may active slow twitch more than fast twitch (McCarthy and Esser, 2007). Siracusa et al. have demonstrated that miRNAs expression is muscle fiber specific and that they have the potential to be new biomarkers of slow or fast muscle damage induced by exercise (Siracusa et al., 2018).

Muscle-specific miRNAs display different responses to eccentric versus concentric mode of contractions (Ebbeling and Clarkson, 1989). Uphill running requires high-energy expenditure or greater muscular activity, while downhill running as a training method to increase the number of sarcomeres is considered as highly strenuous for the muscle-skeletal structures (Malm et al., 2004). It has been suggested muscle damage is usually induced by maximal and submaximal eccentric contractions, but it can also be observed when a high volume of eccentric/concentric contractions are performed, due to the eccentric contractions per se (Eston et al., 1995) or because of metabolite accumulation that may lead to stress and impairment of the muscle fibers, elicit varied muscle adaptation and regenerative responses during the post-exercise period (Appell et al., 1992; Qaisar et al., 2016). In fact, sarcomere genesis was shown to take place in eccentrically trained animals (Butterfield et al., 2005), which can potentially influence muscle performance and flexibility or improve the protective effects against muscle damage (Vogt and Hoppeler, 2014).

Generally, some miRNAs are seen as playing key roles during myogenesis, e.g., miR-1/miR-206 or miR-133, while others likely constitute a kind of muscle property “fine-tuner,” including, e.g., miR-208a/b, which influences muscle performance by myosin switching (Horak et al., 2016). More importantly, previous studies have shown that miR-208b and miR-499 play redundant roles in the specification of muscle fiber identity by activating slow and repressing fast myofiber gene programs (van Rooij et al., 2009), and intracellular miR-499 has been shown to regulate expression of mitochondrial proteins (Wang et al., 2014). In the present study, of six myomiRs investigated, only miR-499 level increased in gastrocnemius after uphill exercise and still elevated 48 h after exercise. Liu et al. found that miR-499 plays an important role in muscle fiber transition and mck-miR-499 transgenic mice derived greater endurance capacity than wild type mice (Liu et al., 2016). It is possible that exercise-induced miR-499 elevation may be involved in the mechanism to couple muscle fiber type and mitochondrial function. The biomechanics, different muscle contraction patterns, and physiological responses have important implications for miRNA expression.

In addition, the time-course recovery of selected six myomiRs in cardiac muscles was similar for both groups, regardless of whether uphill or downhill running was performed. Given that the motor unit recruitment patterns and/or structural differences for muscle regions, it is likely that these myomiRs were not affected.

Thus, these differences in miRNA responses during muscle regions during and following an uphill and/or downhill exercise bout likely appear to be a novel specific adaptive signal within these fibers. The effects of the different mechanical and metabolic factors that would contribute to miRNA responses do not occur at the same time. However, the participation of specific MyomiRs in each one of these processes and the mechanisms involved is still under investigation.

The discovery of cell-free miRNAs in serum, plasma, and other body fluids has yielded an invaluable potential source of non-invasive biomarkers for physical activity and exercise (Baggish et al., 2011). Previous studies have found that some muscle-specialized miRNA levels change in circulation based on the exercise modality, such as endurance (Baggish et al., 2011, 2014; Aoi et al., 2013; Banzet et al., 2013; Gomes et al., 2014; Mooren et al., 2014; Cui et al., 2016; Danese et al., 2018; Ramos et al., 2018), strength (Cui et al., 2017; D’Souza et al., 2017), and sprint exercise (Cui et al., 2015; Denham et al., 2018) or sports (Li et al., 2018). For example, the plasma miR-1, miR-133a, miR-133b, and miR-208b in human were not affected by non-damaging exercise uphill walking, but significantly increased during early recovery of damaging exercise downhill walking, and muscle-related miRNAs miR-181 and miR-214 primarily responded to an uphill exercise (Banzet et al., 2013). It has been suggested that many types of exercise have been shown to produce muscle damage. Both fast twitch and slow twitch fibers may be injured. Damage occurs predominantly in the type I fibers of animals and in the type II fibers of humans. It may be attributed to the type and severity of exercise (Ebbeling and Clarkson, 1989). Individual work periods are much longer in animal studies than in human studies (Ebbeling and Clarkson, 1989). More importantly, the fiber-type specific biomarkers of muscle damage in circulating miRNAs have been found. The plasma miR-133b-3p and miR-434 are related to fast-fiber specific biomarkers, and miR-206-3p is a robust indicator of slow-fiber damage (Siracusa et al., 2018). It is likely that skeletal muscle is comprised of different fiber types that are the basis of muscle plasticity in response to various functional demands.

In the present work, only the miR-133 level did not show a response following uphill running. Although the selected miRNA levels increased, the time-course of dynamic changes over short periods of time was different between the uphill and the downhill exercise. At present, during exercise and recovery, the origins and destinations of miRNAs in circulation are largely unknown. Given the regional differences of fiber properties within muscles in rats (Mao et al., 1992), and based on our results, i.e., that the increase in miR-499 levels in plasma is accompanied by increases in muscle, we speculated that during exercise, these miRNAs in plasma are released from muscle. Although the other miRNAs did not show an increase in the selected muscles, a previous study found that Drosha, Dicer, and Exportin-5, as well as miR-1, miR-133a, and miR-133b, were up-regulated in human skeletal muscle 3 h following an acute bout of moderate-intensity endurance cycling (Russell et al., 2013). In addition, previous studies have not found that a qualitative agreement in the response pattern of intramuscular and circulating miRNA expression within 4 h post-exercise following a single bout of high-intensity resistance exercise (D’Souza et al., 2017). However, it is likely that the resistance exercise-associated increase or decrease in some c-miRNA changes is longer lived and peaks later in the untrained state than in the trained state (Cui et al., 2017).

Because large variations in fiber-type distribution can be found within a muscle or within different parts, and different muscle parts experience diverse exercise load following uphill versus downhill exercise, these increased miRNA levels in plasma are likely from other muscles or stress-susceptible fibers and may reflect the dynamic changes of whole muscle miRNA, suggesting that these different responses may reflect the muscle damage/specificity of exercise adaptations.

Moreover, in biological fluids, miRs are protected against degradation by association with their composition/cargo (Ortiz-Quintero, 2016). Acute exercise-induced exosome release and uptake in circulation are hypothesized to mediate organ crosstalk to promote systemic adaptation to exercise (Aoi, 2014; Mooren et al., 2014). At present, although the exact molecular regulatory mechanisms of muscle-specific miRNA turnover or adaptation during exercise and recovery are unknown, emerging evidence suggests that exercise has a large impact on the biogenesis of exosomes, which are released into circulation during exercise and localize in the liver (Whitham et al., 2018). The exosomes, which carry miRNAs, RNAs, and proteins, play important roles in tissue crosstalk during exercise, which can exert systemic biological effects (Safdar and Tarnopolsky, 2018). In the present study, the six miRNA levels were altered in response to downhill exercise, but not uphill exercise, suggesting that the exercise modality may affect the miRNA levels sorted into exosomes. Furthermore, apart from the exosomes, of the six miRNA levels, only miR-1 and miR-499 were significantly affected by downhill exercise immediately after exercise, only miR-133a was still elevated at 48 h, and only miR-133a was significantly affected by uphill exercise at the immediately after and 48 h recovery time points, suggesting that other encapsulated miRNAs (Arroyo et al., 2011) were also altered by exercise.

Taken together, these different changes appear to relate to alterations in exercise modalities or to be different exercise load of affected muscle parts and perhaps the total fiber number involving exercise. Because large variations in the fiber-type distribution can be found within muscle that is highly adaptable (Mao et al., 1992), our data suggested that the miRNA dynamic changes in tissue and in circulation over short periods of time in acute exercise may play an important role in regulating different contracting skeletal muscles. Subsequently, it is likely that the plasticity of muscle fibers itself was demonstrated by the miRNA flexibility of fiber metabolism as an integrative adaptive mechanism of the whole body.

At present, the different processes of miRNA biogenesis (Nielsen et al., 2010; Russell et al., 2013), packaging (Sapp et al., 2017), secretion (Aoi et al., 2013; Aoi, 2014; Whitham et al., 2018), and uptake/clearance (Aoi et al., 2013; Aoi, 2014) may be important determinants of miRNA effects during exercise and recovery. A better understanding of the cellular and molecular mechanisms of exercise-induced health benefits has important implications for the development of personalized exercise medicine.

## Limitations

There are some limitations in this research. At present, in the miRNA research field, the RNA extraction steps and the qRT-PCR system are not completely unified in different laboratory environments, which affects the further development of miRNA as a biomarker and miRNA function research.

## Conclusion

The results of this study indicate that the miRNA dynamic changes in tissue and circulation over short periods of time in acute exercise may play an important role in regulating different contracting skeletal muscles following specific movement patterns/load variation of different muscle parts. Subsequently, the plasticity of muscle fibers itself was demonstrated by the miRNA flexibility of fiber metabolism as an integrative adaptive mechanism of the whole body for exercise mode. Future studies will be required to identify the potential possible mechanisms responsible for the multiplicity and complexity of cellular pathways and muscle performance involved in these specific exercise-related miRNAs.

## Ethics Statement

The procedures for care and use of animals strictly followed the Guide for the Care and Use of Laboratory Animals published by the US National Institutes of Health (NIH Publication N. 85–23, revised 1996). The protocol was approved by the Ethics Committee of Nanjing University.

## Author Contributions

XY, CZ, XC, and JM designed the research. XY, YZha, JW, and YZhe performed the experiments, analyzed the data, and revised the final manuscript. WL, QL, and QH helped with sample collection, laboratory materials, and reagents. XY and JM wrote the manuscript. All authors approved the final publication of this manuscript.

### Conflict of Interest

The authors declare that the research was conducted in the absence of any commercial or financial relationships that could be construed as a potential conflict of interest.
